# Traditional masculinity ideologies are associated with psychiatric diagnoses in men

**DOI:** 10.1038/s41598-026-45822-5

**Published:** 2026-04-01

**Authors:** Michèle Schneeberger, Ulrike Ehlert, Andreas Walther

**Affiliations:** 1 Department of Clinical Psychology and Psychotherapy, University of Zurich, Zurich, Switzerland; 2 Psychotherapy and Psychotherapy Research (CBT), University of Graz, Elisabethstrasse 32, 8010 Graz, Austria

**Keywords:** Traditional masculinity ideologies, Psychopathology, Psychiatric diagnosis, Diseases, Health care, Medical research, Psychology, Psychology

## Abstract

Conformity to traditional masculinity ideologies (TMIs) has consistently been linked to increased psychopathology. However, little is known about the association with formal psychiatric diagnoses. Cross-sectional data from a randomized controlled trial were analyzed. A total of 317 men (mean age = 31.8, SD = 7.2) completed an online questionnaire assessing conformity to TMIs and underwent the Structured Clinical Interview for DSM-5 (SCID-5) in person. Participants had been pre-screened for depressive symptoms as part of the larger trial, resulting in a sample with an elevated prevalence of depressive symptoms. Inclusion criteria were male sex and gender, age between 25 and 50 years, sufficient German-language skills, and no current or past psychopharmacological or psychological treatment. Overall, 200 participants (63.1%) met the criteria for at least one psychiatric diagnosis, in this pre-screened sample. Logistic regression analyses revealed that higher conformity to TMIs was associated with increased odds of receiving any psychiatric diagnosis (OR = 1.04, 95% CI [1.02, 1.05]), particularly depressive disorders (OR = 1.03, 95% CI [1.01, 1.04]) and substance-related and addictive disorders (OR = 1.03, 95% CI [1.01, 1.05]). No significant associations were found for attention-deficit/hyperactivity disorder or anxiety disorders. Exploratory analyses suggested a significant association between conformity to TMIs and personality disorders, and an initially significant association with sleep–wake disorders that did not survive correction for multiple testing. Higher conformity to TMIs is associated with an increased risk of fulfilling the criteria for a psychiatric diagnosis, especially depressive and substance-related disorders. As such, the study highlights the clinical relevance of including TMIs in the assessment and treatment of men suffering from mental disorders.

*Trial registration*: ClinicalTrials.gov, Identifier: NCT05435222 (Registered: 27.06.2022)

**Supplementary Information:**

The online version contains supplementary material available at 10.1038/s41598-026-45822-5.

## Introduction

According to epidemiological data from Europe and the U.S., around 20% of men suffer from a mental disorder within a 12-month period, most commonly anxiety disorders, alcohol and substance use disorders, and affective disorders^1,2^. Moreover, recent health insurance data from Germany indicate that between 2012 and 2022, the prevalence of diagnosed mental disorders among men increased by 18.3%^3^. This trend might be explained by factors such as increased help-seeking, improved diagnostics, or greater public awareness of mental health problems in men. However, it also draws attention to a possible increase in men’s mental health burden. Compounding this issue, men account for approximately 75% of suicides worldwide^4^, underscoring the critical need to better understand and address men’s mental health.

One gender-specific factor that is increasingly recognized as being crucially related to men’s mental health is the conformity to traditional masculinity ideologies (TMIs). TMIs are socially constructed beliefs and expectations about how men should think, feel, and behave^5,6^. Key dimensions of TMIs include being stoic, prioritizing success and competition, and endorsing casual sexual activity. Additionally, men are often encouraged to use violence to assert dominance or resolve conflict, present themselves as strictly heterosexual, strive to increase their social status and power, and prioritize work above all else. Further dimensions include exerting dominance over women, relying on oneself rather than others, and engaging in risk behaviors^6,7^. While masculinity norms may vary across cultures, TMIs describe a set of widely endorsed standards in Western societies, which shape male identity and socialization^6^.

A growing body of research demonstrates that high conformity to TMIs is associated with elevated psychological distress, reduced help-seeking behavior, and maladaptive coping strategies^8,9^. More specifically, conformity to TMIs has been associated with depression^10–12^, suicidality^13–16^, alcohol and substance abuse^17–20^, anxiety^21^, and aggression, including intimate partner violence^22,23^. These findings suggest that conformity to TMIs may increase men’s vulnerability to the development and exacerbation of mental disorders. However, findings on the association between TMIs and men’s mental health are not entirely consistent. For example, Wong et al.^9^ showed in a meta-analysis that some studies report weak, context-dependent, or null associations, and certain masculinity norms may even be linked to adaptive outcomes such as resilience, perceived control, or social functioning. These mixed findings underscore the complexity of TMIs and highlight the importance of examining their associations with formally assessed psychiatric diagnoses.

As outlined by Pleck^24^,see also^6^), men may experience psychological distress through three distinct types of gender role strain: Discrepancy strain occurs when men fail to meet idealized masculine norms; trauma strain results from early experiences within the process of gender role socialization that are linked to long-term adverse effects; and dysfunction strain occurs when conforming to TMIs leads to negative psychological consequences. Conforming to TMIs often entails suppressing vulnerability, avoiding emotional expression, and prioritizing toughness and independence. As a consequence, many adaptive strategies, such as seeking social support, expressing emotional needs, or engaging in introspection may be perceived as signs of weakness that are incompatible with masculine ideals^25,26^. The restrictive nature of TMIs limits men’s behavioral repertoire, contributing to rigid and often maladaptive responses to psychological distress^27,28^; ^29–31^. These difficulties in managing psychological distress and emotions may lead to an increased vulnerability to and higher prevalence of mental disorders in men with high conformity to TMIs.

Despite substantial research on the association between TMIs and men’s mental health, the relationship between TMIs and formal psychiatric diagnoses has not been sufficiently examined. Previous studies primarily relied on self-reported symptom burden rather than structured clinical assessments, leaving a critical research gap. Guided by theoretical models of gender role strain and prior research suggesting that conformity to TMIs may shape emotional expression, coping strategies, and help-seeking behaviors in ways that could increase men’s vulnerability to psychological distress, the present study examined the association between conformity to TMIs and formally assessed psychiatric diagnoses using the Structured Clinical Interview for DSM-5 (SCID-5).

Based on prior theoretical and empirical work on TMIs and men’s mental health, we hypothesized that higher conformity to TMIs would be associated with increased odds of meeting criteria for at least one psychiatric diagnosis. Second, we hypothesized that higher conformity to TMIs would be particularly associated with depressive disorders and substance-related and addictive disorders. Associations with other diagnostic categories were examined exploratorily due to more limited or inconsistent prior evidence.

## Methods

### Study design and sample

The present study represents a secondary analysis of baseline data from a randomized controlled clinical trial examining a male-specific psychotherapy program for major depressive disorders (MSPP study)^15^. Consequently, recruitment procedures were designed to identify men with elevated depressive symptoms, resulting in a sample enriched for depressive symptomatology rather than representative of the general population. As part of the trial’s inclusion process, participants were pre-screened for depressive symptoms through an online screening questionnaire. Cross-sectional data for the present study were derived from this questionnaire and subsequent clinical assessments. The trial was registered at ClinicalTrials.gov (identifier: NCT05435222) and received ethical approval from the Ethics Committee of the Canton of Zurich (Switzerland): Project-ID: 2022–01,141. The trial registration was completed on 27 June 2022. Because participants were pre-screened for depressive symptoms as part of the larger MSPP study, the resulting sample may not reflect the distribution of psychiatric diagnoses in the general male population.

Participants were recruited through media outlets, paid social media advertisements, and university mailing lists. Interested individuals completed an online screening that collected sociodemographic information, assessed conformity to TMIs, and screened for depressive symptoms to determine eligibility. Both patients and healthy controls were recruited, with screening eligibility based on cut-off scores for depressive symptoms from the Patient Health Questionnaire (PHQ-9) and the Male Depression Risk Scale (MDRS-22): PHQ-9 ≥ 10 or MDRS-22 ≥ 51 for the patient group, and PHQ-9 ≤ 4 and MDRS-22 ≤ 31 for the healthy control group. Additional inclusion criteria were written informed consent, male sex and gender, age 25–50 years, and sufficient German-language proficiency; exclusion criteria included current or previous psychopharmacological or psychological treatment.

**Fig. 1:**
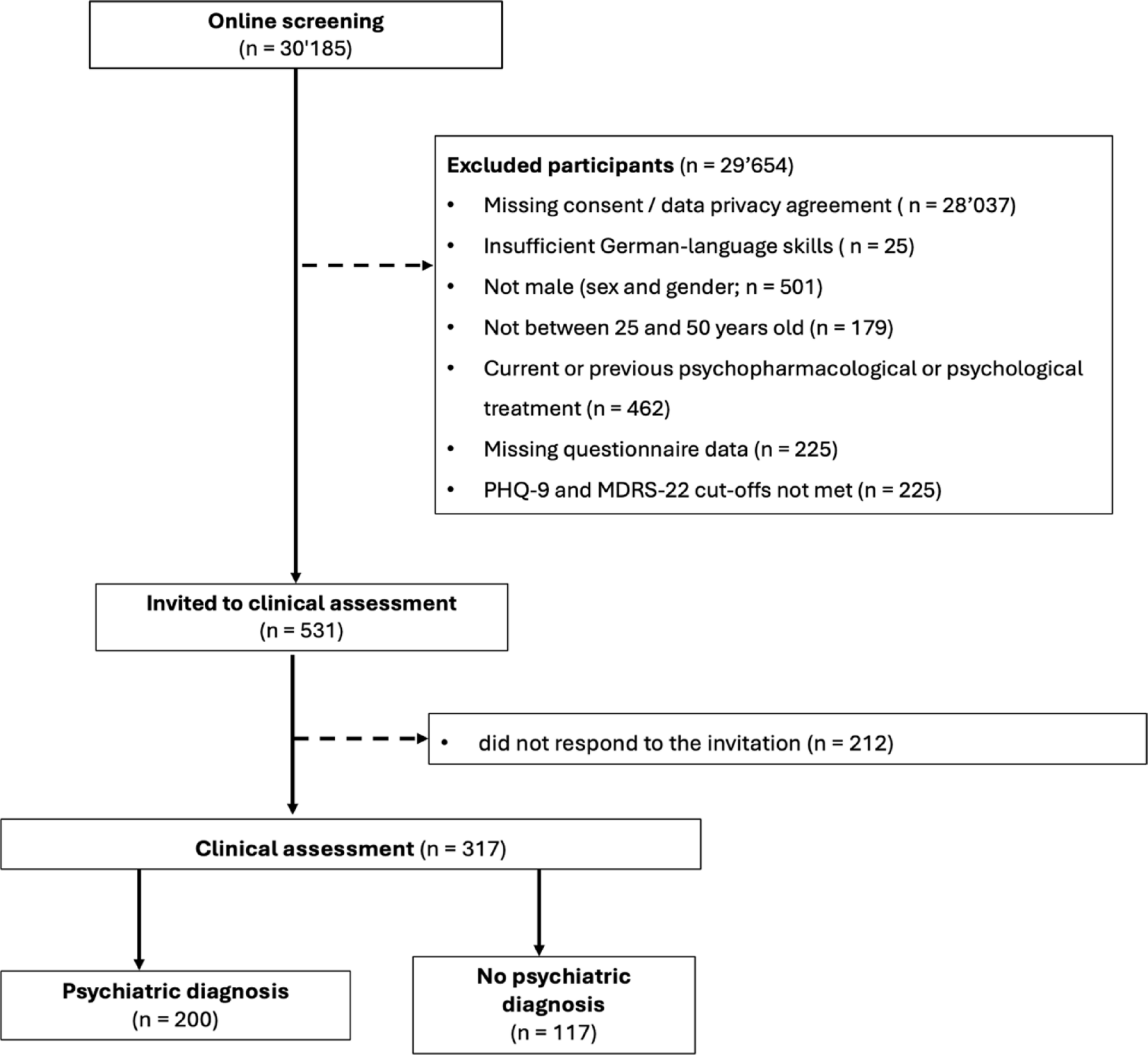
Flowchart Note. n = number of participants.

Of the 30,185 individuals who accessed the online questionnaire, 2148 provided informed consent to participate. Most exclusions occurred because individuals did not complete the consent or data privacy agreement or failed to finish essential screening items. After applying the eligibility criteria, 513 eligible participants were invited to attend a clinical assessment, including the Structured Clinical Interview for DSM-5 (Clinician Version [SCID-5-CV] and Personality Disorder Version [SCID-5-PD]). All interviews were conducted by trained advanced master’s students in psychology. Interviewers received structured training in the administration of the SCID-5, including instruction in diagnostic criteria, supervised practice interviews, and ongoing clinical supervision by experienced clinicians. Diagnostic uncertainties were discussed in regular supervision meetings to ensure consistency and reliability of assessments. A total of 317 men completed the assessment and were included in the present analyses (Fig. 1).

### Instruments

#### Sociodemographic questionnaire

Sociodemographic variables were captured in the online screening questionnaire and included sex assigned at birth, gender identity, age, nationality, ethnicity, highest level of educational attainment, sexual orientation, romantic relationship status, and annual household income.

#### Psychiatric diagnoses

Psychiatric diagnoses were assessed using the German version of the Structured Clinical Interview for DSM—Clinical Version (SCID-5-CV) and Personality Disorders (SCID-5-PD) ^32,33^. The SCID-5-CV systematically evaluates a broad range of major psychiatric disorders, including depressive disorders, bipolar and related disorders, psychotic disorders, substance use disorders, anxiety disorders, obsessive–compulsive and related disorders, trauma- and stressor-related disorders, eating disorders, sleep–wake disorders, somatic symptom and related disorders, and ADHD. The SCID-5-PD was used in the present study to assess the presence of personality disorders across clusters A, B, and C**.** Both interviews are structured, widely used in clinical research and practice, and have demonstrated excellent inter-rater reliability and high specificity^34^. Because each SCID interview was conducted by a single trained interviewer, inter-rater reliability could not be assessed in the present study. Administration typically requires around 90 min, depending on clinical complexity.

#### Conformity to masculine norms inventory—30

The Conformity to Masculine Norms Inventory—30 (CMNI—30;^7^) consists of 30 items assessing conformity to masculine norms on ten subscales: Emotional Control, Winning, Playboy, Violence, Heterosexual Self-Presentation, Pursuit of Status, Primacy of Work, Power over Women, Self-Reliance, and Risk-Taking. Items are rated on a 6-point Likert scale (1 = ”strongly disagree” to 6 = ”strongly agree”). The German-language version used in the present study showed adequate convergent validity and test–retest reliability^35^, with an internal consistency of Cronbach’s α = 0.81 in the present sample. Detailed information on the psychometric properties of the CMNI-30 can be found in Supplementary Table S1.

### Statistical analysis

Statistical analyses were carried out in the R software environment for statistical.

computing and graphics version 4.3.2^36^. For all inferential analyses, the significance level was set at p ≤ 0.05. Stepwise Holm correction was applied to adjust for multiple testing^37^. Primary regression models included age, education, sexual orientation, and relationship status as covariates.

Descriptive statistics and psychometric properties of the CMNI-30 in the present sample were calculated. Group differences in sociodemographic variables and CMNI-30 scores between men with and without psychiatric diagnoses were analyzed using Welch’s two-sample t-tests and χ^2^ tests. Psychometric properties of the CMNI-30 were assessed by internal consistency and distribution of the raw scores (graphically and by skewness and kurtosis; Supplementary Table S1).

A binomial logistic regression analysis using maximum likelihood estimation (MLE) was conducted with TMI (CMNI-30) as predictor and the presence of any psychiatric diagnosis as the binary outcome. Furthermore, separate binomial logistic regressions were performed with TMI (CMNI-30) as predictor and diagnostic categories as outcome variables. To ensure stable estimates and sufficient statistical power, only models with at least 20 cases per diagnostic category were included in the primary analyses. Models with less than 20 cases were treated as exploratory. Diagnostic categories with ≤ 5 cases were excluded from logistic regression analyses entirely, as such small sample sizes lead to unreliable estimates and inflated standard errors^38,39^.

For each model, multicollinearity was assessed using generalized variance inflation factors^40^; all values were below 2, indicating no problematic multicollinearity. Influential data points were examined using Cook’s distance^41^, with no values exceeding the threshold of 1.

## Results

### Descriptive statistics and group differences

**Fig. 2:**
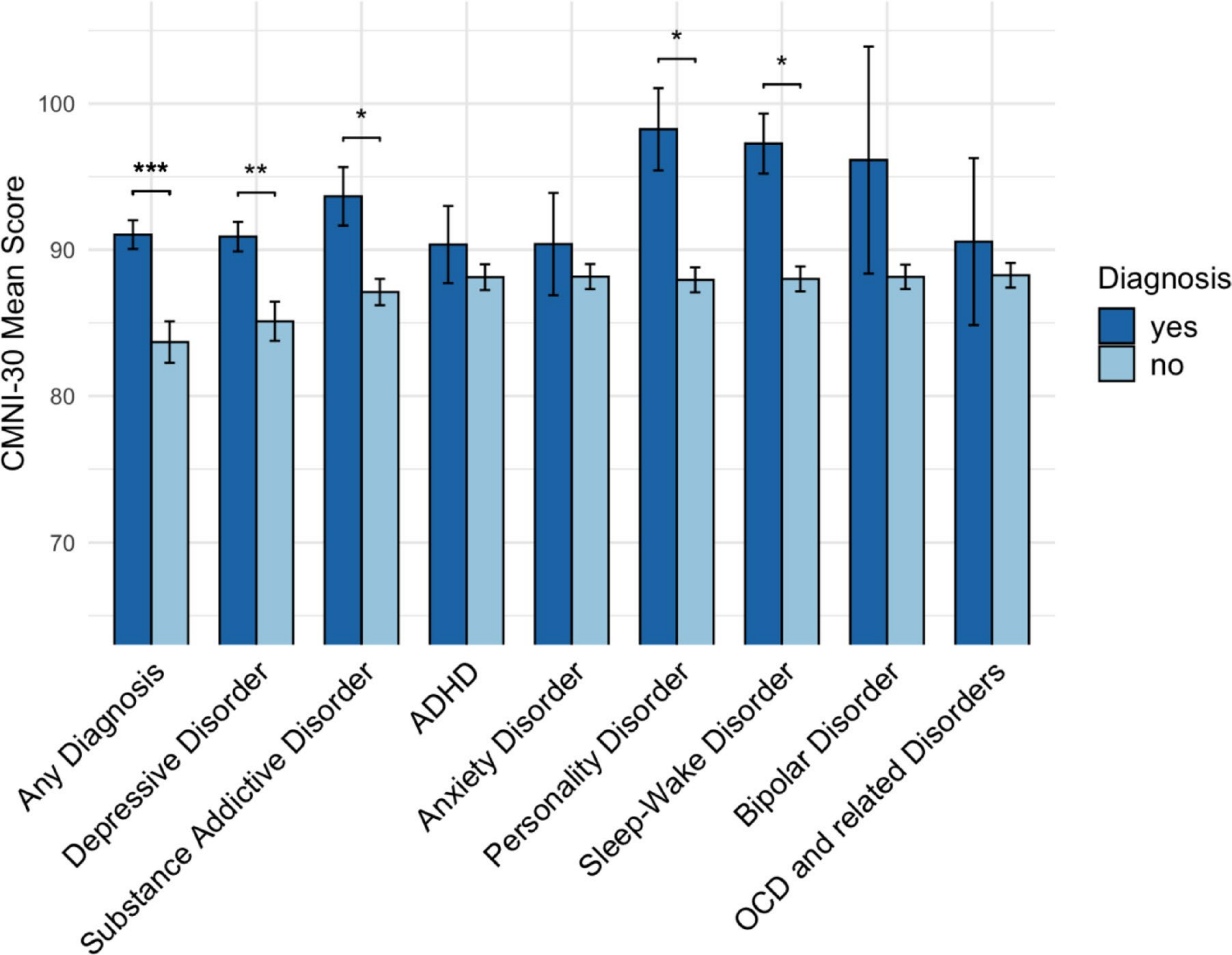
Group Differences in CMNI-30 Mean Scores Between Men With and Without a. Psychiatric Diagnosis *Note.* CMNI-30 = Conformity to Masculine Norms Inventory; ADHD; attention-deficit/hyperactivity disorder; OCD = obsessive-compulsive disorder. *p*-values reflect logistic regression analyses; primary models were covariate-adjusted, whereas exploratory models (personality disorder, sleep-wake disorder, bipolar disorder, OCD and related disorders) were unadjusted due to small sample sizes. Error bars represent standard errors. * *p* < .05. ** *p* < .01. *** *p* < .001

The pre-screened study sample consisted of 317 men, of whom 200 (63.1%) met the criteria for at least one current psychiatric diagnosis (Table 1). Although lifetime information was collected as part of the SCID-5 interview, only current diagnoses were considered in the present analyses. There were no significant differences in sociodemographic variables between men with and without a psychiatric diagnosis. Men with a psychiatric diagnosis showed higher conformity to TMIs compared to those without a psychiatric diagnosis. This association was found across several diagnostic categories, with significant group differences observed for depressive disorders, substance-related and addictive disorders, personality disorders, and sleep–wake disorders (Fig. 2). As shown in Table 2, the most prevalent diagnoses were found in the categories of depressive disorders (n = 187), substance and alcohol use disorders (n = 72), ADHD (n = 28), and anxiety disorders (n = 23). Among the 200 men diagnosed with at least one mental disorder, 89 met the criteria for one diagnosis, 62 for two diagnoses, 34 for three diagnoses, and 15 for four or more diagnoses. The most frequently observed comorbidities were between depressive disorders and substance and alcohol use disorders (n = 51), depressive disorders and ADHD (n = 22), and depressive disorders and anxiety disorders (n = 19).

**Table 1:** Descriptive statistics of the sample.

Variable	Total sample (n = 317)	Psychiatric diagnosis (n = 200)	No psychiatric diagnosis (n = 117)	t / χ^2^ (df)	effect	*p*	*p* (corr.)
Age*,* mean (SD)	31.8 (7.1)	32.1 (7.2)	31.3 (7.2)	− 0.99 (245.21)	0.11	.325	.921
Nationality, n (%)				5.45 (2)	0.13	.066	.393
Swiss	231 (72.9)	137 (68.5)	94 (80.3)				
German	60 (18.9)	43 (21.5)	17 (14.5)				
Other	26 (8.2)	20 (10.0)	6 (5.1)				
Ethnicity, n (%)				0.00 (1)	0.00	1	1
Western/European	296 (93.4)	187 (93.5)	109 (93.2)				
Non− Western	21 (6.6)	13 (6.5)	8 (6.8)				
Education, n (%)				0.06 (1)	.013	.813	1
Tertiary education	221 (69.7)	138 (69.0)	83 (70.9)				
Non− tertiary education	96 (30.3)	62 (31.0)	34 (29.1)				
Sexual orientation, n (%)				Fisher’s exact	0.12	.208	.921
Heterosexual	280 (88.3)	173 (86.5)	107 (91.5)				
Gay	20 (6.3)	15 (7.5)	5 (4.3)				
Bisexual	12 (3.8)	10 (5.0)	2 (1.7)				
Other / not sure	5 (1.6)	2 (1.0)	3 (2.6)				
Romantic relationship, n (%)				1.42 (1)	0.07	.234	.921
Yes	180 (56.8)	108 (54.0)	72 (61.5)				
No	137 (43.2)	92 (46.0)	45 (38.5)				
Annual household income, n (%)				9.14 (2)	0.17	.010*	.072
< 25,000 CHF		37 (18.5)	24 (20.5)				
25,000–75,000 CHF		65 (32.5)	20 (17.10)				
> 75,000 CHF		98 (49.0)	73 (62.4)				
CMNI− 30, mean (SD)	88.33 (14.85)	91.04 (13.9)	83.7 (15.4)	− 4.26 (223.48)	0.51	< .001***	< .001***
Emotional Control	11.36 (3.67)	12.05 (3.61)	10.19 (3.5)	− 4.54 (250.14)	0.52	< .001***	< .001***
Winning	9.05 (2.87)	9.30 (2.91)	8.63 (2.76)	− 2.04 (253.54)	0.23	.043*	.298
Playboy	8.68 (3.90)	9.02 (3.79)	8.09 (4.02)	− 2.02 (231.19)	0.24	.045*	.298
Violence	8.26 (3.75)	8.14 (3.51)	8.45 ((4.13)	0.07 (212.75)	0.08	.493	1
Heterosexual Self− Presentation	6.03 (3.34)	6.10 (3.31)	5.90 (3.40)	− 0.50 (237.63)	0.06	.621	1
Pursuit of Status	11.59 (2.74)	11.69 (2.66)	11.44 (2.89)	− 0.76 (226.65)	0.09	.446	1
Primacy of Work	8.82 (3.22)	9.12 (3.33)	8.31 (2.96)	− 2.25 (266.25)	0.25	.025*	.201
Power over Women	4.95 (2.37)	5.10 (2.41)	4.96 (2.30)	− 1.48 (251.93)	0.17	.140	.702
Self− Reliance	9.78 (3.64)	10.73 (3.32)	8.15 (3.60)	− 6.35 (227.01)	0.76	< .001***	< .001***
Risk− Taking	9.81 (3.31)	9.80 (3.27)	9.84 (3.39)	0.10 (235.85)	0.01	.923	1

n = number of participants; SD = standard deviation; t = t-statistic, χ^2^ = Chi-squared statistic; effect = Cohen’s d for numerical and Cramér’s V for categorical variables; corr. = corrected for multiple testing using the Holm method; CMNI-30 = Conformity to Masculine Norms Inventory—30;

* *p* < .05; ** *p* < .01; *** *p* < .001.

Significant values are in bold.

**Table 2:** Distribution of psychiatric diagnoses (n = 317).

Diagnosis	n (%)
**Depressive disorder**	**187 (59.0)**
Major depressive disorder, single episode	93 (29.3)
Major depressive disorder, recurrent episode	46 (14.5)
Persistent depressive disorder (dysthymia)	40 (12.6)
Other specified depressive disorder or unspecified depressive disorder	8 (2.5)
**Substance-related and addictive disorder**	**72 (22.7)**
Alcohol use disorder	43 (13.6)
Substance use disorder	29 (9.2)
Gambling disorder	–
**Attention-deficit/hyperactivity disorder**	**28 (8.8)**
**Anxiety disorder**	**25 (7.9)**
Panic disorder	8 (2.5)
Agoraphobia	1 (0.3)
Social anxiety disorder	7 (2.2)
Generalized anxiety disorder	7 (2.2)
Separation anxiety disorder	–
Specific phobia	2 (0.6)
**Personality disorder**	**15 (4.7)**
Avoidant	5 (1.6)
Dependent	-
Obsessive–compulsive	6 (1.9)
Paranoid	1 (0.3)
Schizoid	1 (0.3)
Schizotypal	–
Histrionic	–
Narcissistic	1 (0.3)
Borderline	–
Antisocial	1 (0.3)
**Sleep–wake disorder**	**13 (4.1)**
Insomnia	8 (2.5)
Hypersomnia	5 (1.6)
**Bipolar and related disorder**	**7 (2.2)**
Bipolar I disorder	3 (1.0)
Bipolar II disorder	3 (1.0)
Other specified bipolar and related disorder or unspecified bipolar and related disorder	1 (0.0)
**Obsessive–compulsive and related disorder**	**7 (2.2)**
Obsessive–compulsive disorder	5 (1.6)
Trichotillomania	2 (0.6)
Dermatillomania	–
Body dysmorphic disorder	–
**Somatic symptom and related disorder**	**5 (1.6)**
Illness anxiety disorder	–
Somatic symptom disorder	5 (1.6)
**Eating disorder**	**4 (1.26)**
Anorexia nervosa	–
Bulimia nervosa and binge eating disorder	4 (1.3)
Avoidant/restrictive food intake disorder (arfid)	–
**Post-traumatic stress disorder**	**2 (0.6)**
**Any diagnosis**	**200 (63.1)**
1 diagnosis	89 (28.1)
2 diagnoses	62 (19.6)
3 diagnoses	34 (10.7)
4 or more diagnoses	15 (4.7)

n = number of participants

Main psychiatric disorders are indicated in bold.

### Logistic regression analyses examining the association between conformity to TMIs and the likelihood of diagnoses

Logistic regression analyses (Table 3) revealed that higher conformity to TMIs was associated with increased odds of fulfilling the criteria for at least one psychiatric diagnosis (*p* < 0.001, OR = 1.04, 95% CI [1.02, 1.05]). This corresponds to an approximate 4% increase in the odds of receiving a psychiatric diagnosis for each one-point increase on the CMNI-30. At the subscale level, higher scores on the subscales Emotional Control (*p* = 0.008, OR = 1.10, 95% CI [1.02, 1.19]), Playboy (*p* = 0.015, OR = 1.08, 95% CI [1.01, 1.16]), and Self-Reliance (*p* < 0.001, OR = 1.13, 95% CI [1.10, 1.29]) were associated with greater odds of receiving at least one psychiatric diagnosis. However, after correcting for multiple testing, only the association with the Self-Reliance subscale remained statistically significant (see Supplementary Table S2). Conformity to TMIs significantly predicted the likelihood of fulfilling the criteria for a depressive disorder (*p* < 0.001, OR = 1.03, 95% CI [1.01, 1.04]) and a substance-related and addictive disorder (*p* = 0.003, OR = 1.03, 95% CI [1.01, 1.05]). These associations remained statistically significant after adjusting for sociodemographic covariates and correcting for multiple testing. No significant associations were found between conformity to TMIs and diagnoses of ADHD or anxiety disorders.

**Table 3:** Logistic Regression Analyses with TMIs (CMNI-30) and Covariates.

Variable	β (SE)	OR^a^		CI	*p*-value	*p* adj
**(A) Outcome: any diagnosis**						
Intercept	− 3.21 (0.93)	0.04		[0.01, 0.25]	> .001***	.003**
CMNI-30	0.03 (0.01)	1.04		[1.02, 1.05]	> .001***	> .001***
Age	0.02 (0.02)	1.02		[0.98, 1.05]	.292	.944
Education	0.06 (0.27)	1.06		[0.63, 1.79]	.823	.944
Sexual Orientation	0.48 (0.40)	1.61		[0.73, 3.53]	.236	.944
Relationship	0.19 (0.25)	1.21		[0.74, 1.97]	.458	.944
Omnibus statistics: χ^2^(5) = 21.88, p value > .001***, R^2^ = 9.1%, AIC = 407.6, BIC = 430.1						
**(B) Outcome: depressive disorders**						
Intercept	− 3.150 (0.91)	0.04		[0.01, 0.25]	> .001***	.003**
CMNI-30	0.026 (0.01)	1.03		[1.01, 1.04]	> .001***	.004**
Age	0.029 (0.02)	1.03		[1, 1.06]	.090	.360
Education	0.109 (0.26)	1.12		[0.68, 1.84]	.671	.671
Sexual Orientation	0.547 (0.38)	1.73		[0.82, 3.64]	.150	.451
Relationship	0.251 (0.24)	0.24		[0.8, 2.06]	.299	.598
Omnibus statistics: χ^2^(5) = 18.05, p value = .003**, R^2^ = 7.4%, AIC = 407.6, BIC = 430.1						
**(C) Outcome: substance-related and addictive disorders**						
Intercept	− 3.778 (1.19)	0.02		[0, 0.23]	.001**	.009**
CMNI-30	0.027 (0.01)	1.03		[1.01, 1.05]	.003**	.016*
Age	− 0.015 (0.02)	0.98		[0.94, 1.03]	.490	1
Education	0.244 (0.32)	1.28		[0.69, 2.36]	.439	1
Sexual Orientation	− 0.264(0.48)	0.77		[0.30, 1.98]	.583	1
Relationship	0.528 (0.30)	1.70		[0.94, 3.06]	.079	.317
Omnibus statistics: χ^2^(5) = 14.14, p value = .015*, R^2^ = 7.1%, AIC = 302.5, BIC = 325.1						
**(D) Outcome: attention-deficit / hyperactivity disorder**						
Intercept	− 3.435 (1.52)		0.03	[0, 0.64]	.024*	.144
CMNI-30	0.010 (0.01)		1.01	[0.98, 1.04]	.229	1
Age	0.005 (0.03)		1.00	[0.95, 1.06]	.867	1
Education	− 0.085 (0.44)		0.92	[0.39, 2.19]	.848	1
Sexual Orientation	0.811 (0.50)		2.25	[0.84, 6.01]	.105	.527
Relationship	-0.093 (0.41)		0.91	[0.41, 2.04]	.822	1
Omnibus statistics: χ^2^(5) = 3.03, p value = .696, R^2^ = 2.1%, AIC = 198.3, BIC = 220.9						
**(E) Outcome: anxiety disorders**						
Intercept	-2.829 (1.66)		0.06	[0, 1.53]	.089	.531
CMNI-30	0.012 (0.014)		1.01	[0.98, 1.04]	.417	1
Age	− 0.019 (0.03)		0.98	[0.92, 1.05]	.564	1
Education	0.145 (0.46)		1.16	[0.47, 2.86]	.753	1
Sexual Orientation	− 0.319 (0.77)		0.73	[0.16, 3.25]	.677	1
Relationship	− 0.451 (0.46)		0.46	[0.26, 1.58]	.329	1
Omnibus statistics: χ^2^(5) = 2.09, p value = .836, R^2^ = 1.6%, AIC = 174.9, BIC = 197.4						

SE = standard error; *p* adj. = *p*-values adjusted for multiple testing using the Holm method; R^2^ = Nagelkerke’s (Cragg and Uhler) pseudo-R^2^; AIC = Akaike information criterion; BIC = Bayesian information criterion. Reference category is non-tertiary education, non-heterosexual, and single. Significant associations are highlighted in bold.

^a^ Displayed coefficients are z-standardized.

* *p* < .05. ** *p* < .01. *** *p* < .001.

### Exploratory logistic regression analyses

Exploratory logistic regression analyses (Table 4) showed that conformity to TMIs significantly predicted the likelihood of diagnosis with a personality disorder (*p* = 0.010, OR = 1.05, 95% CI [1.01, 1.09]) and a sleep–wake disorder (*p* = 0.023, OR = 1.04, 95% CI [1, 1.08]). However, the latter association did not remain significant after correction for multiple testing (see Supplementary Table S3 . No significant associations were found between conformity to TMIs and diagnoses of bipolar disorder or obsessive–compulsive and related disorders.

**Table 4:** Exploratory logistic regression analyses with TMIs (CMNI-30).

Variable	β (SE)	OR^a^	CI	*p-*value
**(A) Outcome: personality disorders**				
Intercept	− 7.41 (1.92)	0.00	[0, 0.03]	< .001***
CMNI-30	0.05 (0.02)	1.05	[1.01, 1.09]	.010 *
Omnibus statistics: χ^2^(1) = 5.39, p value = .020*, R^2^ = 6.1%, AIC = 100.7, BIC = 108.2				
**(B) Outcome: sleep–wake disorders**				
Intercept	− 7.06(1.97)	0.00	[0, 0.04]	> .001***
CMNI-30	0.04 (0.02)	1.04	[1, 1.08]	.023 *
Omnibus statistics: χ^2^(1) = 4.01, p value = .045*, R^2^ = 4.8%, AIC = 95.5, BIC = 103.1				
**(C) Outcome: bipolar and related disorders**				
Intercept	− 6.99(2.41)	0.00	[0, 0.10]	.004**
CMNI-30	0.04 (0.03)	1.04	[0.99, 1.09]	.081
Omnibus statistics: χ^2^(1) = 1.92, p value = .166, R^2^ = 3.2%, AIC = 69.3, BIC = 76.8				
**(D) Outcome: obsessive–compulsive and related disorders**				
Intercept	− 4.45 (2.06)	0.01	[0, 0.66]	.031*
CMNI-30	0.01 (0.02)	1.01	[0.97, 1.06]	.324
Omnibus statistics: χ^2^(1) = 0.21, p value = .649, R^2^ = 0.3%, AIC = 85.6, BIC = 93.2				

SE = standard error;* p* adj. = *p*-values adjusted for multiple testing using the Holm method; R^2^ = Nagelkerke’s (Cragg and Uhler) pseudo-R^2^; AIC = Akaike information criterion; BIC = Bayesian information criterion. Significant associations are highlighted in bold.

^a^ Displayed coefficients are z-standardized.

* *p* < .05. ** *p* < .01. *** *p* < .001.

A supplementary sensitivity and specificity analysis was conducted using the CMNI-30 total score and the self-reliance subscale to predict the presence of any psychiatric diagnosis. Results, including positive and negative predictive values as well as overall accuracy, are reported in the Supplementary Tables S4 and S5.

## Discussion

### Summary of results

The present study investigated the association between conformity to TMIs and psychiatric diagnoses among men using the Structured Clinical Interview for DSM-5 (SCID-5). Given the pre-screening, a majority of participants met diagnostic criteria for at least one psychiatric disorder; nevertheless, men with a psychiatric diagnosis showed significantly higher conformity to TMIs compared to those without a diagnosis. Logistic regression analyses confirmed that higher conformity to TMIs was associated with increased odds of receiving at least one psychiatric diagnosis. At the subscale level, self-reliance emerged as the most robust predictor of psychiatric diagnoses. Among the diagnostic categories examined, conformity to TMIs significantly predicted the diagnosis of depressive disorders and substance-related and addictive disorders, even after controlling for sociodemographic covariates and correcting for multiple testing. No significant associations were found for ADHD or anxiety disorders. Exploratory analyses further revealed a significant association between TMIs and personality disorders, while the initially observed association with sleep–wake disorders was no longer significant after correction for multiple testing.

### Integration of findings

Our finding that higher conformity to TMIs was associated with increased odds of receiving at least one formal psychiatric diagnosis in men extends previous research that reported an association of TMIs with unfavorable mental health outcomes (e.g.^9^). While previous studies largely relied on self-reported symptom measures, the present findings reveal that conformity to TMIs is also associated with formal psychiatric diagnoses based on a structured clinical interview.

At the subscale level, self-reliance emerged as the most consistent predictor of any psychiatric diagnosis, highlighting the psychological costs associated with conformity to TMIs that discourage emotional disclosure and help-seeking^25,26^. Notably, self-reliance was the only TMI dimension that remained statistically significant after correction for multiple testing, suggesting that reluctance to seek support and strong emphasis on autonomy may represent a particularly central mechanism linking TMIs to psychiatric diagnoses in men. Importantly, social isolation and reduced help-seeking are well-established risk factors for mental health problems across populations and are influenced by a wide range of interpersonal, structural, and psychological factors beyond TMIs alone. For instance, socioeconomic conditions, personality traits, stigma related to mental illness, and prior experiences with healthcare systems may also shape help-seeking behavior and perceived self-reliance^42–44^. Thus, conformity to TMIs should not be interpreted as the sole driver of these processes but rather as one sociocultural factor that may amplify existing vulnerabilities to isolation and untreated psychological distress among men.

An alternative explanation should also be considered. Rather than conformity to TMIs increasing vulnerability to mental health problems, experiencing psychological distress may itself reinforce self-reliance or compensatory conformity to TMIs, for example as a coping strategy to preserve identity, autonomy, or avoid stigma related to mental health difficulties. However, this bidirectional relationship cannot be disentangled within the present cross-sectional design.

Beyond self-reliance, initial associations were also observed for the subscales Emotional Control and Playboy. While these associations did not survive correction for multiple testing, both reflect additional TMI dimensions that may be associated with experiences of dysfunction strain^24^ over time. For instance, emotional control may be associated with lower emotional awareness and reduced use of adaptive coping strategies, and the playboy norm, which focuses on external validation through sexual success, may fbe linked to an unstable, performance-based self-worth and emotional detachment. Both dimensions, when internalized by young men, may therefore may therefore be linked to a reduced sense of self-awareness (e.g. through a lack of connection to one’s own feelings and emotions) and a reduced sense of self-worth (e.g. through numerous superficial, often sexual encounters hindering the establishment of long-lasting nurturing relationships), in turn increasing men’s vulnerability to mental disorders.

Regarding specific diagnostic categories, conformity to TMIs was significantly associated with depressive disorders as well as substance-related and addictive disorders. These findings align with previous research linking TMI conformity to both depression (e.g.,^9,11,45^) and alcohol or substance abuse (e.g^20^). Several potential mechanisms underlying these associations have been discussed. For instance, rigid conformity to norms like self-reliance and emotional control may be associated with psychological strain such as isolation or untreated distress^24^,as a consequence, psychological burden may remain unacknowledged or untreated, increasing the risk of clinical depression over time^25,26,43^. Simultaneously, if the depressive symptoms are themselves in conflict with men’s internalized TMIs, secondary burden might result^46^. The sense of failing to meet standards of stoicism, emotional control, and toughness may exacerbate feelings of shame, failure, or identity conflict^47,48^. Thus, depression may not only be more likely in men who conform strongly to TMIs, but may also be more complex in its presentation, reinforcing cycles of distress and maladaptive coping strategies.

Furthermore, men may perceive substance use as a means to manage distress without violating gender role expectations. For men in particular, alcohol use is often culturally accepted, if not implicitly encouraged, as an expression of autonomy or toughness (Iwamoto et al., 2011). The high comorbidity between depressive and substance-related disorders in the present sample should be interpreted in light of the study’s pre-screening procedure for depressive symptoms, which likely contributed to an elevated base rate of depressive disorders and associated comorbidities. Nevertheless, this pattern may additionally reflect a maladaptive dynamic in which psychological distress is managed through substance use in ways that conform to TMIs, while failing to address underlying emotional needs. Taken together, these results underscore the clinical importance of addressing conformity to TMIs in assessment and treatment planning for men who present with depressive disorders and substance-related and addictive disorders^29,49–51^.

In contrast, no significant association emerged between conformity to TMIs and anxiety disorders. This finding may reflect a conceptual mismatch between TMIs and the typical presentation of anxiety disorders. TMIs promote toughness and risk-taking, while anxiety disorders are characterized by avoidance of feared situations or internal states^52,53^. Men who strongly conform to TMIs may be more inclined to suppress anxiety-related behaviors or to confront their fears, thereby exposing themselves to potentially fearful situations and leading to fear extinction learning^54^. Importantly, neurobiological differences between men and women regarding fear extinction should also be considered^55^. At the same time, high conformity to TMIs that discourage emotional vulnerability may lead men to underreport anxiety symptoms, or to express distress through externalizing behaviors such as irritability or anger, which could reduce the likelihood that anxiety symptoms are recognized or diagnosed^21,56^. These dynamics may help to explain the observed lack of association between TMIs and anxiety diagnoses in the present study. Additionally, because participants were pre-screened for depression symptoms as part of the larger trial, men with predominantly anxiety disorders but without comorbid depressive symptoms may have been underrepresented. This sampling characteristic could partly account for the absence of a significant association between TMIs and anxiety diagnoses. More broadly, conformity to TMIs may also influence diagnostic processes themselves. TMIs may contribute to gendered symptom expression and potential diagnostic biases, including underrecognition of internalizing disorders in men^48,57,58^.

Furthermore, no significant association was observed between a diagnosis of ADHD and conformity to TMIs. ADHD is typically characterized by early emerging neurodevelopmental disturbances in executive function, attention regulation, and impulse control, largely driven by genetic and biological factors^59^. In contrast, conformity to TMIs reflects a sociocultural process through which individuals internalize culturally normative beliefs about masculinity (gender role socialization;^60,61^). Although gender role socialization begins in childhood, it may not meaningfully interact with the early neurodevelopmental pathways associated with ADHD. Moreover, empirical research specifically examining the relationship between TMIs and ADHD remains scarce, underscoring the need for further investigation into how gender norms might shape the expression or perception of neurodevelopmental disorders in men.

While exploratory in nature, the observed association between conformity to TMIs and personality disorders may point to deeper identity conflicts or interpersonal difficulties associated with rigid conformity to TMIs. However, these findings should be interpreted with considerable caution given the small number of cases for specific personality disorders in the present sample, which limits the stability and generalizability of these exploratory results. For instance, dimensions such as self-reliance and emotional control may be linked to obsessive–compulsive personality disorder, as individuals who show high conformity to these norms may experience excessive control over their emotions and actions, leading to perfectionism, rigidity, and difficulties in forming flexible, adaptive relationships. Similarly, the emphasis on winning and the pursuit of status in TMIs could be associated with narcissistic personality traits^62^. The drive for success and external validation inherent in these TMI dimensions could foster narcissistic traits, including an inflated sense of self-importance, a constant need for admiration, and a lack of empathy. Furthermore, the initial findings regarding sleep–wake disorders could reflect the impact of TMI-related stress on physiological regulation and sleep quality. Although these associations did not withstand correction for multiple testing, they warrant further investigation in larger samples.

The present findings also have implications for clinical assessment and routine mental health practice. In everyday clinical settings, psychiatric diagnoses are often based on unstructured or semi-structured clinical assessments rather than fully standardized diagnostic interviews. Our use of the SCID-5 interview provides a comparatively rigorous diagnostic reference point, suggesting that associations between conformity to TMIs and psychiatric diagnoses are not limited to self-report symptom measures but extend to clinically established diagnoses. At the same time, the association of TMIs with symptom expression, emotional disclosure, and help-seeking may complicate diagnostic decision-making in routine clinical practice. Our findings therefore underscore the value of gender-sensitive assessment approaches, including careful exploration of externalizing or atypical symptom presentations in men.

Moreover, the results support growing calls for integrating standardized diagnostic procedures where feasible and for incorporating gender-sensitive psychotherapeutic approaches that explicitly address TMIs^15,63,64^. Such approaches may improve diagnostic accuracy, treatment engagement, and ultimately clinical outcomes among male patients. Finally, the present study adds to research on clinical decision-making in men’s mental health by highlighting how sociocultural gender norms may shape not only symptom development but also their recognition and interpretation in clinical contexts^28,47,57^.

### Limitations and future directions

Several limitations should be considered when interpreting the present findings. First, the cross-sectional design limits causal inference: While higher conformity to TMIs was associated with psychiatric diagnoses, the directionality of this relationship remains unclear. It is possible that rigid conformity to TMIs may precede and contribute to the development or maintenance of psychological distress through mechanisms such as emotional suppression, reduced help-seeking, or maladaptive coping. Conversely, experiencing mental health difficulties may also shape how men relate to masculinity norms, for example by reinforcing rigid self-reliance or compensatory conformity to TMIs in response to perceived vulnerability. Additionally, more complex associations, including potential mediating processes (e.g., social isolation, maladaptive coping) cannot be disentangled within the present cross-sectional design.

Second, the small number of cases for some diagnostic categories (e.g., personality disorders n = 15, sleep–wake disorders n = 13, bipolar disorders n = 7, obsessive–compulsive and related disorders n = 7), may have reduced the statistical power and limited the detection of associations for less prevalent disorders. In particular, the exploratory analyses involving sleep–wake and personality disorders should be replicated in larger samples to confirm their robustness.

Third, the sample composition may restrict generalizability. Participants were pre-screened for depressive symptoms as part of a larger trial, which likely influenced the distribution of diagnoses. Accordingly, the observed prevalence rates cannot be generalized to the broader male population, where 12-month prevalence estimates for mental disorders are typically around 20% in epidemiological studies^1^^,^^2^. Moreover, men who chose to participate may have been more interested or engaged in mental health topics. Previous research suggests that men with high conformity to TMIs are generally more reluctant to seek help^8,10,65^, potentially resulting in an underrepresentation of this group in the present study.

Fourth, conformity to TMIs was assessed using a self-report measure (CMNI-30), which may be influenced by social desirability biases, self-presentation concerns, or limited introspective awareness. This is particularly relevant in research on gender norms, where endorsement of TMIs may itself be shaped by normative expectations or reluctance to disclose socially sensitive attitudes.

Fifth, although interviewers were not aware of participants’ CMNI-30 scores, they were not formally blinded to the broader study context. While diagnoses were based on structured SCID-5 criteria, the absence of full blinding may have introduced some potential for expectancy bias cannot be fully excluded.

Lastly, clinical interviews were conducted by a highly trained research team with gender-sensitive and particularly male-specific expertise, which may not reflect the diagnostic standards and practices in routine care. In many everyday clinical settings, diagnoses are often based on less standardized assessments conducted under time constraints and with varying levels of training in gender-sensitive mental health assessment. Consequently, the associations observed in the present study may not translate directly to routine clinical practice. Nevertheless, our findings highlight the potential importance of increasing awareness of masculinity-related influences on symptom expression and help-seeking among clinicians, even when fully standardized diagnostic interviews are not feasible.

## Conclusion

The present study provides evidence that higher conformity to TMIs is associated with increased odds of receiving formal psychiatric diagnoses in men, particularly depressive disorders and substance-related and addictive disorders. Our findings underscore the relevance of TMIs as a clinically meaningful construct in the assessment of men’s mental health. Given their association with a wide range of psychiatric outcomes, TMIs may serve as an important psychosocial factor to consider in diagnostic processes. Future research should continue to examine how TMIs are linked to symptom presentation and diagnostic pathways, and how preventive and therapeutic approaches can be adapted to address the mental health risks associated with rigid conformity to TMIs^51,66^. Incorporating gender-sensitive frameworks into clinical practice may improve diagnostic accuracy and promote treatment engagement among male patients.

## Supplementary Information

Below is the link to the electronic supplementary material.

Supplementary Material 1

## Data Availability

The data and code used for the present study will be made available by the corresponding author upon reasonable request.
