# Supplementary material for: Traditional masculinity ideologies are associated with psychiatric diagnoses in men
Source: Sci Rep. 2026 Apr 1;16:15326. doi: 10.1038/s41598-026-45822-5 (PMC13181103; doi:10.1038/s41598-026-45822-5)
Supplement: Supplementary file 1 — Supplementary Material 1 [file 41598_2026_45822_MOESM1_ESM.pdf]

Traditional Masculinity Ideologies Are Associated with Psychiatric Diagnoses in Men: Evidence from the Structured Clinical Interview for DSM-5 with 317 Men

Michèle Schneeberger, M.Sc.[1]; Ulrike Ehlert, PhD [1]; Andreas Walther, PhD[2*]

[1] Department of Clinical Psychology and Psychotherapy, University of Zurich, Zurich, Switzerland
[2] Psychotherapy and Psychotherapy Research, University of Graz, Graz, Austria
* Corresponding author

Supplementary Materials

Content

**S1 Table**	*Psychometric Properties of the CMNI-30*	page 2
**S2 Table**	*Exploratory Logistic Regression Analyses with TMI (CMNI-30) and Covariates*	page 3
**S3 Table**	*Exploratory Logistic Regression Analyses with TMI (CMNI-30) Subscales*	page 4
**S4 Table**	*Sensitivity Analyses Including Income Instead of Education as Covariate.*	page 5

Table S1. *Psychometric Properties of the CMNI-30*

	n_{items}	α	Mean (SD)	Range	Skewness	Kurtosis
**CMNI-30**	30	.81	2.94 (0.50)	[1; 6]	0.25	-0.08
Emotional Control	3	.89	3.79 (1.22)		-0.08	-0.91
Winning	3	.65	3.02 (0.96)		0.24	-0.45
Playboy	3	.84	2.89 (1.3)		0.24	-0.79
Violence	3	.56	2.75 (1.25))		0.32	-0.79
Heterosexuality	3	.91	2.01 (1.11)		1.27	0.97
Pursuit of Status	3	.61	3.86 (0.91)		-0.39	0.38
Primacy of Work	3	.75	2.94 (1.07)		0.28	-0.56
Power over Women	3	.77	1.65 (0.79)		1.53	2.38
Self-Reliance	3	.77	3.26 (1.21)		0.08	-0.68
Risk-Taking	3	.85	3.27 (1.10)		0.07	-0.63

Note. n_{items} = number of items; α = Cronbach's alpha; SD = standard deviation; CMNI-30 = Conformity to Masculine Norms Inventory – 30

Table S2. *Exploratory Logistic Regression Analyses with TMI (CMNI-30) and Covariates*

Variable	β (SE)	OR[a]	CI	*p* value	*p* adj.
(A) Outcome: personality disorder					
Intercept	-9.773 (2.65)	0.00	[0, 0.01]	**<.001*****	**.001****
CMNI-30	0.050 (0.021)	1.05	[1.01, 1.09]	**0.008 ****	**0.038 ***
Age	0.058 (0.037)	1.06	[0.98, 1.14]	.124	.496
Education	0.484 (0.615)	1.62	[0.49, 5.41]	.432	1
Sexual Orientation	-0.363 (1.078)	1.70	[0.08, 5.76]	.736	1
Relationship	-0.456 (0.642)	1.63	[0.18, 2.23]	.478	1
Omnibus statistics: $\chi^2(5) = 9.12$, p value = .105, R^2 = 10.3%, AIC = 105.0, BIC = 127.6					
(B) Outcome: sleep-wake disorder					
Intercept	-8.57 (2.67)	0.00	[0, 0.04]	**.001****	**0.008****
CMNI-30	0.04 (0.02)	1.04	[1, 1.09]	**.031 ***	.155
Age	0.04 (0.04)	1.04	[0.95, 1.13]	.411	1
Education	0.24 (0.65)	1.27	[0.35, 4.57]	.714	1
Sexual Orientation	-16.42 (1706.63)	0.00	[0, inf]	.992	1.00
Relationship	0.87 (0.65)	2.38	[0.66, 8.6]	.184	.737
Omnibus statistics: $\chi^2(5) = 9.23$, p value = .100, R^2 = 11.0%, AIC = 98.3, BIC = 120.9					
(C) Outcome: Bipolar Disorder					
Intercept	-8.45 (3.19)	0.00	[0, 0.11]	**.008****	**.048***
CMNI-30	0.04 (0.03)	1.04	[0.99, 1.09]	.057	.285
Age	0.04 (0.05)	1.04	[0.94, 1.14]	.449	1
Education	0.53 (0.79)	1.70	[0.36, 8.02]	.506	1
Sexual Orientation	0.38 (1.12)	1.46	[0.16, 13.15]	.735	1
Relationship	-1.62 (1.10)	0.20	[0.02, 1.71]	.141	.569
Omnibus statistics: $\chi^2(5) = 6.23$, p value = .285, R^2 = 10.2%, AIC = 73.0, BIC = 95.6					
(D) Outcome: Obsessive-Compulsive and Related Disorders					
Intercept	-3.92 (2.59)	0.02	[0, 3.21]	.131	.786
CMNI-30	0.01 (0.02)	1.01	[0.97, 1.06]	.298	1
Age	-0.02 (0.05)	0.98	[0.89, 1.08]	.699	1
Education	0.10 (0.73)	1.10	[0.26, 4.58]	.894	1
Sexual Orientation	0.84 (0.83)	2.33	[0.46, 11.77]	.307	1
Relationship	-0.56 (0.73)	0.57	[0.14, 2.40]	.444	1
Omnibus statistics: $\chi^2(5) = 1.74$, p value = .883, R^2 = 2.4%, AIC = 92.1, BIC = 114.7					

Note. SE = standard error; *p* adj. = *p*-values adjusted for multiple testing using the Holm method; R^2 = Nagelkerke's (Cragg and Uhler) pseudo-R^2; AIC = Akaike information criterion; BIC = Bayesian information criterion. Reference category is non-tertiary education, non-heterosexual, and single. Significant associations are highlighted in bold.

[a] Displayed coefficients are z standardized.

* $p < .05$. ** $p < .01$. *** $p < .001$

Table S3. *Exploratory Logistic Regression Analyses with TMI (CMNI-30) Subscales*

**Variable**	**β (SE)**	**OR[a]**	**CI**	***p*-value**	***p* adj.**
(A) Outcome: any diagnosis					
Intercept	-3.30 (0.90)	0.04	[0.01, 0.22]	**<.001*****	**.003****
Emotional Control	0.10 (0.04)	1.10	[1.02, 1.19]	**.008****	.072
Winning	0.03 (0.05)	1.04	[0.90, 1.04]	.259	1
Playboy	0.08 (0.04)	1.08	[1.01, 1.16]	**.015***	.120
Violence	-0.04 (0.04)	0.97	[0.90, 1.04]	.330	1
Heterosexual self-presentation	-0.03 (0.04)	0.97	[0.89, 1.05]	.463	1
Pursuit of status	0.02 (0.05)	1.03	[0.93, 1.13]	.315	1
Primacy of work	0.04 (0.04)	1.04	[0.96, 1.14]	.161	1
Power over women	0.03 (0.07)	0.07	[0.90, 1.17]	.345	1
Self-reliance	0.18 (0.04)	1.19	[1.10, 1.29]	**<.001*****	**<.001*****
Risk-taking	-0.02 (0.04)	0.98	[0.91, 1.07]	.680	1
Omnibus statistics: $\chi^2(10)$ = 53.35, p value = <.001***, R^2 = 21.2%, AIC = 386.1, BIC = 427.5					

Note. SE = standard error; *p* adj. = *p*-values adjusted for multiple testing using the Holm method; R^2 = Nagelkerke's (Cragg and Uhler) pseudo-R^2; AIC = Akaike information criterion; BIC = Bayesian information criterion. Reference category is non-tertiary education, non-heterosexual, and single. Significant associations are highlighted in bold.

[a] Displayed coefficients are z standardized.

* *p* < .05. ** *p* < .01. *** *p* < .001

Table S4. *Sensitivity Analyses Including Income Instead of Education as Covariate.*

Variable	β (SE)	OR[a]	CI	*p*-value	*p* adj.
(A) Outcome: any diagnosis					
Intercept	-3.81 (0.98)	0.02	[0, 0.15]	**>.001*** **	**>.003** **
CMNI-30	0.04 (0.01)	1.04	[1.02, 1.06]	**>.001*** **	**>.001*** **
Age	0.05 (0.02)	1.05	[1.01, 1.09]	.054	0.32
Income	-0.06 (0.20)	0.94	[0.91, 0.98]	**.011***	0.06
Sexual Orientation	0.49 (0.40)	1.64	[0.74, 3.61]	.446	1
Relationship	0.05 (0.26)	1.06	[0.64, 1.75]	.837	1
Omnibus statistics: $\chi^2(5)$ = 31.4, p value >.001***, R^2 = 12.9%, AIC = 398.1, BIC = 420.6					
(B) Outcome: depressive disorders					
Intercept	-3.931 (1.54)	0.02	[0, 0.40]	**.005****	**.026***
CMNI-30	0.026 (0.01)	1.03	[1.01, 1.04]	**>.001*** **	**.002****
Age	0.026 (0.02)	1.03	[0.99, 1.06]	.090	.443
Income	0.082 (0.15)	1.09	[0.68, 1.84]	.671	1
Sexual Orientation	0.075 (0.16)	1.08	[0.79, 1.47]	.150	1
Relationship	0.207 (1.13)	1.23	[0.95, 1.60]	.299	.443
Omnibus statistics: $\chi^2(5)$ = 17.80, p value = .003**, R^2 = 7.3%, AIC = 429.8, BIC = 452.3					
(C) Outcome: substance-related and addictive disorders					
Intercept	-4.039 (1.22)	0.02	[0, 0.23]	**>.001*** **	**.002****
CMNI-30	0.029 (0.01)	1.03	[1.01, 1.05]	**.003****	**.009****
Age	-0.001 (0.03)	1.00	[0.95, 1.05]	.960	1
Income	-0.034 (0.03)	0.96	[0.92, 1.02]	.189	0.580
Sexual Orientation	-0.259(0.48)	0.77	[0.30, 1.99]	.583	1
Relationship	0.443 (0.30)	1.56	[0.86, 2.83]	.079	.317
Omnibus statistics: $\chi^2(5)$ = 15.42, p value = .008**, R^2 = 7.7%, AIC = 301.2, BIC = 323.8					
(D) Outcome: attention-deficit / hyperactivity disorder					
Intercept	-3.756(1.53)	0.02	[0, 0.64]	**.014***	.085
CMNI-30	0.011 (0.01)	1.01	[0.98, 1.04]	.422	1
Age	0.022 (0.02)	1.02	[0.96, 1.09]	.492	1
Income	-0.034 (0.35)	0.97	[0.90, 1.03]	.328	1
Sexual Orientation	0.838 (0.50)	2.31	[0.86, 6.19]	.095	.475
Relationship	-0.161 (0.42)	0.85	[0.38, 1.93]	.700	1
Omnibus statistics: $\chi^2(5)$ = .03, p value = .545, R^2 = 2.8%, AIC = 197.3, BIC = 219.9					
(E) Outcome: anxiety disorders					
Intercept	-2.7'7(1.69)	0.09	[0, 1.82]	.109	.652
CMNI-30	0.012 (0.014)	1.01	[0.98, 1.04]	.411	1
Age	-0.024 (0.04)	0.98	[0.91, 1.05]	.527	1

Income	0.007 (0.33)	1.01	[0.94, 1.07]	.826	1
Sexual Orientation	-0.327 (0.76)	0.76	[0.16, 3.23]	.669	1
Relationship	-0.445 (0.46)	0.47	[0.26, 1.60]	.341	1
Omnibus statistics: $\chi^2(5) = 2.04$, p value = .843, R^2 = 1.6%, AIC = 174.9, BIC = 197.5					

Note. SE = standard error; *p* adj. = *p*-values adjusted for multiple testing using the Holm method; R^2 = Nagelkerke's (Cragg and Uhler) pseudo-R^2; AIC = Akaike information criterion; BIC = Bayesian information criterion. Reference category is non-tertiary education, non-heterosexual, and single. Significant associations are highlighted in bold.

[a] Displayed coefficients are z-standardized.

* *p* < .05. ** *p* < .01. *** *p* < .001.
